# Relationship between physical exercise and subjective wellbeing in university students: the chain mediation role of self-identity and self-esteem

**DOI:** 10.3389/fpsyg.2025.1637779

**Published:** 2025-09-03

**Authors:** Lihua Yao, Kelei Guo, Feng Guo, Dong Li, Yanying Liu, Jun Xiang

**Affiliations:** ^1^School of Physical Education, Shangqiu Normal University, Shangqiu, China; ^2^School of Physical Education and Health, Zhaoqing University, Zhaoqing, China; ^3^School of Physical Education, Guangdong Technology College, Zhaoqing, China

**Keywords:** physical exercise, university students, subjective wellbeing, self-esteem, self-identity

## Abstract

**Objective:**

Subjective wellbeing, a fundamental concept in positive psychology, encompasses an individual’s evaluation of their life satisfaction and the balance between positive and negative emotions, thus reflecting personal perceptions and thoughts about life. This research aimed to analyze the interplay between physical exercise and subjective wellbeing among university students, with a particular emphasis on the mediating roles of self-identity and self-esteem. The study investigates the correlation between physical exercise and subjective wellbeing, further exploring how physical exercise affects self-identity and subsequently impacts subjective wellbeing. It also examines how self-identity influences self-esteem and the mediation role of self-esteem between self-identity and subjective wellbeing. This research constructed a chained mediation model encompassing physical exercise, self-identity, self-esteem, and subjective wellbeing to elucidate their interactions, ultimately proposing targeted exercise strategies to enhance subjective wellbeing through university physical education programs and personal exercise regimens.

**Methods:**

Utilizing physical exercise scales, self-identity scales, self-esteem scales, and subjective wellbeing questionnaires, this study conducted a survey among 913 university students. Data analysis proceeded via Pearson correlation analysis, structural equation modeling, and bias-corrected percentile bootstrap methods.

**Results:**

Physical exercise showed a positive correlation with subjective wellbeing (*r* = 0.49), exhibiting significant direct pathways from physical exercise to subjective wellbeing (*β* = 0.43, *p* < 0.01, CI [0.36, 0.50]). Physical exercise was also positively correlated with self-identity (*β* = 0.37, *p* < 0.01, CI [0.30, 0.44]) and self-esteem (*β* = 0.36, *p* < 0.01, CI [0.30, 0.43]), with self-identity positively influencing self-esteem (*β* = 0.31, *p* < 0.01, CI [0.23, 0.39]), both of which positively impact subjective wellbeing (self-identity: *β* = 0.17, *p* < 0.01, CI [0.11, 0.23]; self-esteem: *β* = 0.18, *p* < 0.01, CI [0.11, 0.25]). Furthermore, self-identity and self-esteem significantly mediated the relationship between physical exercise and subjective wellbeing through multiple pathways.

**Conclusion:**

These findings highlight a significant positive correlation between physical exercise and subjective wellbeing. Both self-identity and self-esteem serve as independent and sequential mediators in this relationship, underlining the complex interdependencies in the chain mediation model.

## 1 Introduction

With the development of modern society and the increasing attention to mental health, the psychological wellbeing and subjective wellbeing of college students have become a focal point of societal concern. As the main body of higher education, college students face multiple pressures, such as academic challenges, employment prospects, and social relationships, while simultaneously exploring and shaping their self-identity. The psychological health of college students is related to individual growth and development and has significant implications for social harmony and progress. The 2023 China Mental Health Report indicates that mental health issues among students are becoming increasingly prominent and are showing a trend toward younger age groups. The proportions of anxiety and depression risks among college students are 45.28 and 21.48%, respectively. In this process, subjective wellbeing serves as an important indicator of college students’ quality of life, which relates to individual mental health and reflects the quality of higher education and social development. Therefore, exploring effective ways to enhance the subjective wellbeing of college students is of enormous practical significance.

Positive psychologists argue that happiness is a positive emotional experience, and an individual’s sense of happiness does not depend on the hardships they face but rather on how they emotionally respond to their reality. Currently, the most widely accepted concept of subjective wellbeing among scholars is that it refers to individuals’ subjective evaluations of their life quality (Kugbey et al., 2018). Mental health is a comprehensive state that encompasses emotional, cognitive, and behavioral functions, while subjective wellbeing specifically refers to an individual’s perception and emotional evaluation of their life. The two are related but not equivalent. For instance, people with high subjective wellbeing may still face mental health risks due to physical illness, while those with low wellbeing may maintain basic psychological functions through social support. This study focuses on subjective wellbeing because it more directly reflects college students’ satisfaction and emotional experience with their current life, which is more in line with the “immediate feedback” characteristics of physical exercise (such as the release of endorphins after exercise). Physical exercise has been shown to have a significant impact on improving individuals’ mental health. Through physical activity, individuals can improve their physical health and effectively enhance their mental wellbeing. However, the specific psychological pathways through which physical exercise influences the subjective wellbeing of college students have not been fully addressed. Self-identity and self-esteem are essential components of an individual’s psychological structure. The mechanism of action between physical exercise and subjective wellbeing requires further exploration. To explain the relationship between the two, this study constructed a mediation model and aimed to deepen the understanding of how physical exercise affects the subjective wellbeing of college students through empirical research.

This study overcomes the limitations of traditional single-mediating research and, for the first time, incorporates self-identity and self-esteem into a dynamic progressive framework. It reveals the complete mechanism by which physical exercise influences subjective wellbeing through a dual resource transformation path of “individual identity construction (self-identity) → self-worth assessment (self-esteem).” By integrating the resource preservation theory with the social cognition theory, it is proposed that physical exercise not only directly enhances psychological resources (self-identity) but also forms a “dual channel” of resource accumulation by improving the self-evaluation system (self-esteem), providing a more systematic theoretical framework for understanding the generation of positive psychological outcomes.

### 1.1 **Physical exercise and** subjective wellbeing

Physical exercise refers to planned, regular, and repetitive physical activities aimed at enhancing physical fitness and improving both physical and mental health, occurring through the interaction between an individual’s conditions and the external environment (Mileva and Zaidell, 2022). Researchers have widely acknowledged that physical exercise promotes mental health. Its role in enhancing the psychological wellbeing and life skills development of college students has attracted significant academic attention. Moreover, physical exercise has advantages in developing college students’ psychological resources and cultivating personality traits. Thus, sports theory research should focus on the mental health development of students (Cronin et al., 2018).

Subjective wellbeing is a key topic discussed in the field of positive psychology in recent years. It refers to individuals’ subjective evaluations of their overall quality of life and serves as a standard for measuring an individual’s psychological development and mental health. It is an important indicator of mental health levels (Veenhoven, 2011). The level of subjective wellbeing reflects, to some extent, an individual’s mental health status. Understanding the intrinsic relationship between physical exercise and college students’ subjective wellbeing is of immense significance for enhancing mental health education among college students. Malli and others found that physical exercise influences happiness by up to 40%, and students who engage in campus sports activities experience enhanced subjective wellbeing levels (Malli and Yildizhan, 2018). There is a significant positive correlation between physical exercise and subjective wellbeing in college students, meaning that physical exercise has a significant positive predictive effect on subjective wellbeing (Liao et al., 2023). David and colleagues, in a cross-sectional study of adolescent health-promoting behaviors and subjective wellbeing, found that physical exercise is a unique predictor of subjective wellbeing. The more physical exercise adolescents engage in, the higher their levels of subjective wellbeing (Smith et al., 2022). Therefore, we propose Hypothesis 1: Physical exercise has a positive impact on the subjective wellbeing of college students.

### 1.2 The mediating role of self-identity

Self-identity refers to the awareness of oneself as distinct from and coexisting with others, with a sense of continuity and stability. It involves the integration and coordination of an individual’s internal state and external environment, aligning the ideal self and the real self within the personality structure in harmony and consistency (Fachrunisa and Riyono, 2023). The sense of self-identity among college students pertains to an individual’s recognition of who they are within the school environment and maintaining a sustained and stable identification with their perceived self (Zhao and Zhao, 2012).

The resource conservation theory emphasizes that individuals cope with stress and pursue positive development by acquiring, retaining, and utilizing resources. Physical exercise requires individuals to invest initial resources such as time, energy, and physical strength. Individuals will not consume these resources meaninglessly unless they expect to gain greater benefits. Individuals, through achieving their exercise goals, transform their initial investment into internalized psychological resources (self-identity). Those with high self-identity are more likely to view exercise as “resource acquisition” rather than “consumption,” thereby maintaining a long-term exercise habit and forming a virtuous cycle of enhanced self-identity (Hobfoll, 1989). Previous research demonstrated that physical exercise and self-identity are significantly related. This is evident from the fact that exercise intensity, duration, and frequency are significantly associated with current self-investment, past risks, and future intentions and desires for self-investment (Delgado-Floody et al., 2022). Positive correlation between physical exercise and sense of self-identity implies that the higher the level of physical exercise among college students, the stronger their sense of self-identity (Cao and Luo, 2024). Additionally, domestic scholars have conducted extensive research on the relationship between self-identity and subjective wellbeing among college students. They showed that self-identity is significantly related to subjective wellbeing. From the perspective of individual development, the university phase is a period of rapid psychological changes for college students. The formation of self-identity can enhance an individual’s self-efficacy, improve their adaptability, and subsequently influence their psychological wellbeing, thereby enhancing subjective wellbeing (Guo et al., 2024).

Relevant research results confirmed this viewpoint, which shows a significant positive correlation between self-identity and subjective wellbeing among college students. Further, self-identity has a significant positive predictive effect on college students’ subjective wellbeing (Cui et al., 2021). When the development of self-identity is disrupted, students may experience inner conflicts and contradictions, leading to increased negative emotional experiences and a tendency to develop a negative self-concept. This leads to difficulties in realizing the self-concept and unclear self-role cognition, which negatively impacts the overall subjective wellbeing of students (Du et al., 2017). Conversely, when self-identity develops well, students can recognize, view, and accept themselves dialectically and objectively, improving their adaptability and regulation abilities, which enhances their subjective wellbeing (Zhang and Chen, 2018). Therefore, we proposed Hypothesis 2: Self-identity mediates the relationship between physical exercise and subjective wellbeing among college students.

### 1.3 The mediating role of self-esteem

Self-esteem is an individual’s positive cognitive evaluation of their worth. It is an internal cognitive process related to self-information that occurs during the pursuit of personal value realization (Kim et al., 2022). This concept holds a pivotal position in the field of psychology as it forms an essential foundation of the self-structure. Self-esteem is regarded as a personality variable with the ability to predict emotional states and life changes, occupying a central role in personality psychology. For college students, who are in a critical stage of personal development, self-esteem is an important component of self-awareness. Extensive theoretical frameworks and empirical findings have been accumulated in the in-depth study of its influencing factors.

Self-determination theory emphasizes the intrinsic motivation and self-determination tendency of human behavior. It holds that individuals have three fundamental psychological needs: the need for autonomy, the need for competence, and the need for relationships. When these needs are met, individuals will experience higher intrinsic motivation, better psychological states, and a sense of happiness. Physical exercise offers individuals the opportunity to meet these basic psychological needs, which in turn affects self-esteem and subjective wellbeing. Physical exercise enhances an individual’s self-esteem level by meeting their basic psychological needs, improving their body image, and providing a successful experience. High self-esteem, as a mediating variable, influences an individual’s subjective wellbeing through the transmission of positive self-awareness, enhancing the ability to cope with stress and setbacks, and promoting the formation of good interpersonal relationships. Previous research has shown a significant positive correlation between physical exercise and overall self-esteem. The greater the intensity of physical exercise and the longer the duration of participation, the higher the individual’s level of self-esteem (Rodrigues et al., 2022). A relevant study found through correlational analysis that there is a positive relationship between physical exercise and self-esteem, with a high correlation coefficient. The two factors mutually reinforce each other; the higher the level of physical exercise among college students, the higher their self-esteem (Kim and Ahn, 2021). Self-esteem in adolescents, as an important internal factor influencing subjective wellbeing, can significantly predict the level of an individual’s subjective wellbeing (Pazzaglia et al., 2020). A study found that subjective wellbeing is significantly positively correlated with self-esteem. Self-esteem significantly and positively predicts subjective wellbeing; the higher the level of self-esteem, the stronger the individual’s subjective wellbeing (Ouyang et al., 2020).

Another study investigating the relationship between physical exercise and subjective wellbeing in older adults demonstrated that self-esteem partially mediates this relationship. Social support and self-esteem act as chain mediators in the relationship between physical exercise and subjective wellbeing among older adults (Chen and Gao, 2023). Research has shown that physical exercise can directly enhance college students’ subjective wellbeing and also indirectly improve it by alleviating interpersonal relationship issues and enhancing self-esteem (Bang et al., 2020).

In summary, we propose Hypothesis 3: Self-esteem mediates the relationship between physical exercise and subjective wellbeing among college students.

### 1.4 The chain mediating role of self-identity and self-esteem

Self-identity and self-esteem both pertain to the individual’s self-level and are significant domains in psychological research. Self-identity essentially involves an in-depth exploration and answer of the questions “Who am I?” and “What kind of person do I hope to become in the future?” It reveals an individual’s cognition and expectations regarding their self-identity and goals. In contrast, self-esteem is an individual’s subjective evaluation of their overall status, reflecting their perception and feelings about their self-worth. Therefore, there must be a close connection between self-identity and self-esteem.

Currently, most research on self-identity and self-esteem focuses on their state relationships, aiming to uncover the connections and influences between the two. A relevant study found that the level of self-esteem varies under different identity states or stages. Individuals in a state of completed self-identity exhibit higher levels of self-esteem (Kim, 2022). Among college students, self-identity and self-esteem are significantly positively correlated. The higher the sense of self-identity, the higher the level of self-esteem, and an individual’s self-esteem level can be accurately and effectively predicted by self-identity (Colak et al., 2023). Previous research demonstrated that physical exercise can influence subjective wellbeing through the complete mediating role of self-esteem and also through the chain mediating roles of body image and self-esteem (Shang et al., 2021).

Furthermore, based on the positive correlations between physical exercise and self-identity, physical exercise and self-esteem, as well as self-identity, self-esteem, and subjective wellbeing, we confidently propose Hypothesis 4: Self-identity and self-esteem play a chain mediating role in the relationship between physical exercise and subjective wellbeing.

**Figure 1:**
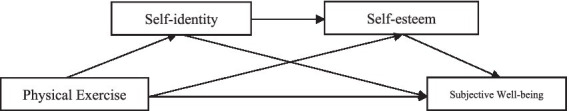
Conceptual framework.

In summary, this study constructs a chain mediating model of the relationship between physical exercise and subjective wellbeing. Specifically, from the perspective of physical exercise, it validates the relationship mechanism between physical exercise and subjective wellbeing among Chinese college students. It explores the roles of self-identity and self-esteem within this model (Figure 1).

## 2 Methods

### 2.1 Participants

The stratified cluster sampling method was used to randomly select five classes from each grade to distribute questionnaires. A total of 989 students participated in the questionnaire. After excluding invalid questionnaires, 913 valid questionnaires were collected, with an effective recovery rate of 92.32%. The age range of the subjects was 19.61 ± 1.05 years, ranging from 18 to 22 years. The sample included 445 men and 468 women. There are 250 freshmen, 239 sophomores, 244 juniors, and 180 seniors.

This study was conducted following the Declaration of Helsinki. All participants were informed of the purpose and nature of the study and provided signed informed consent forms. Participation was voluntary, and confidentiality was assured for the students. The principal investigators were all professionally trained physical education students. During the testing process, consent was obtained from both teachers and students.

### 2.2 Measures

In this study, to ensure the scientific validity and effectiveness of the measurement tools, all the scales adopted underwent a rigorous compilation or revision process, and the verification work was specifically carried out for the group of Chinese college students. All the scales demonstrated high internal consistency reliability, good content validity, and structural validity among the Chinese college student population. They could accurately and stably measure the corresponding research variables, providing a solid guarantee for obtaining high-quality data in this study and fully demonstrating that these scales have high adaptability to the Chinese college student population.

#### 2.2.1 Physical exercise

Physical exercise was assessed using the Physical Activity Scale developed by Chen et al. (2006) and revised by Wu (2016). This scale consists of eight items, encompassing two dimensions: commitment to physical exercise and persistence in physical exercise. All items are rated on a 5-point Likert scale (1 = “Strongly disagree” to 5 = “Strongly agree”), with the total score representing the participant’s level of physical activity. A higher total score indicates a higher level of physical activity. This scale has been proven to have high applicability among Chinese college student populations (Jiang et al., 2018). In this study, the scale demonstrated a Cronbach’s *α* of 0.83.

#### 2.2.2 Self-identity

Self-identity was measured using the Self-Identity Questionnaire developed by Kato (1983) and revised by Zhang (2000). The questionnaire is divided into three dimensions: “Past Crises,” “Current Self-Investment,” and “Future Self-Investment Desires,” comprising a total of 12 items. A six-point Likert scale was used (1 = “Completely Not,” 6 = “Completely Yes”), with the total score representing the participant’s level of self-identity. Higher scores indicate a higher degree of self-identity. This scale has been demonstrated to have high applicability among Chinese college student populations (Dou et al., 2016). In this study, the scale exhibited a Cronbach’s *α* of 0.88.

#### 2.2.3 Self-esteem

Self-esteem was measured using the Rosenberg Self-Esteem Scale (Jordan, 2020), which was revised by Wang et al. (2019). The goal is to evaluate the self-esteem of college students. The scale consists of 10 items and utilizes a 4-point Likert scale (1 = “Completely Disagree,” 4 = “Completely Agree”). The theoretical score range of the scale is 10 to 40 points, with higher scores indicating higher levels of self-esteem. Previous research has shown that this scale has good applicability within the Chinese college student population (Xie et al., 2019). In this study, the scale demonstrated a Cronbach’s *α* coefficient of 0.79.

#### 2.2.4 Subjective wellbeing

Subjective wellbeing was measured using the Subjective wellbeing Scale developed by Campbell et al. (1976) and revised by Wang et al. (1999). The questionnaire consists of nine items, divided into the Emotional Index (8 items, weighted at 1) and the Life Satisfaction Index (1 item, weighted at 1.1). A 7-point Likert scale was used (1 = “Low,” 7 = “High”), with higher scores indicating higher levels of wellbeing. Research has demonstrated that this questionnaire has good applicability among Chinese college student populations (Peng, 2022). In this study, the scale exhibited a Cronbach’s α coefficient of 0.90.

### 2.3 Statistical analysis

Descriptive analysis, Pearson correlation analysis, independent samples t-test, one-way ANOVA, multiple regression analysis, and chain mediation analysis using the Process macro were conducted using SPSS 26.0. A significance level of *p* < 0.05 was set to determine statistical significance. Age and gender were controlled as covariates in the analyses.

## 3 Results

### 3.1 Common method bias test

Participants responded to the questionnaire items through self-report. Since all subjects were from the same university, there was a potential for common method bias to affect the statistical results. To this end, the questionnaire survey was conducted anonymously, clearly informing the participants that the data was only for research and completely anonymous, reducing social approval bias. Moreover, a common method bias test was carried out for all measurement items on the four scales in this study. Therefore, Harman’s single-factor test was conducted on the collected data. The results indicated that there were eight factors with eigenvalues greater than 1, and the first factor explained 24.74% of the variance, which is below the critical threshold of 40%. Thus, it was concluded that there was no serious issue with common method bias.

### 3.2 Descriptive statistics and correlation analysis

The correlation analysis results (Table 1) showed that physical exercise was significantly correlated with self-identity, self-esteem, and subjective wellbeing in all pairwise comparisons. Notably, the correlations among self-identity, self-esteem, and subjective wellbeing are particularly noteworthy, suggesting that enhancing self-identity and self-esteem among college students may contribute to improving subjective wellbeing. These findings provide preliminary support for our hypotheses.

**Table 1:** Correlation analysis of variables.

Variable	*M*	*SD*	1	2	3	4
Physical exercise	28.30	6.17	1			
Self-identity	52.66	8.06.	0.36**	1		
Self-esteem	38.60	4.97	0.44**	0.43**	1	
Subjective wellbeing	37.03	7.28	0.47**	0.36**	0.39**	1

*N* = 913; **p < 0.01.

### 3.3 Mediation effect analysis

Based on the non-parametric percentile bootstrap method proposed by Hayes (2018), mediation effects were tested using the PROCESS (Version 3.3) macro Model 6 with 5,000 bootstrap samples and a 95% confidence interval (CI), controlling for age and gender (Table 2).

1. We examined the direct path between physical exercise and subjective wellbeing among college students. The results showed that physical exercise was positively correlated with subjective wellbeing (*r* = 0.49). The direct path was significant, *β* = 0.43, *p* < 0.01, CI [0.36, 0.50], thereby supporting Hypothesis 1.
2. We examined the mediating roles of self-identity and self-esteem between physical exercise and subjective wellbeing (see Figure 1). The results indicated that physical exercise was positively correlated with self-identity, *β* = 0.37, *p* < 0.01, CI [0.30, 0.44], and self-identity was positively correlated with subjective wellbeing, *β* = 0.17, *p* < 0.01, CI [0.11, 0.23], thereby supporting Hypothesis 2. Physical exercise was positively correlated with self-esteem, *β* = 0.36, *p* < 0.01, CI [0.30, 0.43], and self-esteem was positively correlated with subjective wellbeing, *β* = 0.18, *p* < 0.01, CI [0.11, 0.25], thereby supporting Hypothesis 3.
3. Self-identity and self-esteem were positively correlated, *β* = 0.31, *p* < 0.01, CI [0.23, 0.39], thereby supporting Hypothesis 4.

**Table 2:** Analysis of regression relationships among variables.

Effect	Item	Effect	SE	*t*	*p*	LLCI	ULCI
Direct effect	Physical exercise ⇒ Subjective wellbeing	0.28	0.04	8.00	<0.01	0.21	0.35
Indirect effect process	Physical exercise ⇒ Self-identity	0.37	0.36	10.33	<0.01	0.30	0.44
	Physical exercise ⇒ Self-esteem	0.36	0.34	10.59	<0.01	0.30	0.43
	Self-identity ⇒ Self-esteem	0.31	0.04	7.58	<0.01	0.23	0.39
	Self-identity ⇒ Subjective wellbeing	0.17	0.03	5.43	<0.01	0.11	0.23
	Self-esteem ⇒ Subjective wellbeing	0.18	0.04	5.02	<0.01	0.11	0.25
Total effect	Physical exercise ⇒ Subjective wellbeing	0.43	0.03	12.35	<0.01	0.36	0.50

All variables in the model have been standardized, as shown below; LLCI is the lower limit of the 95% interval of estimated value, and ULCI is the upper limit of the 95% interval of estimated value.

**Figure 2:**
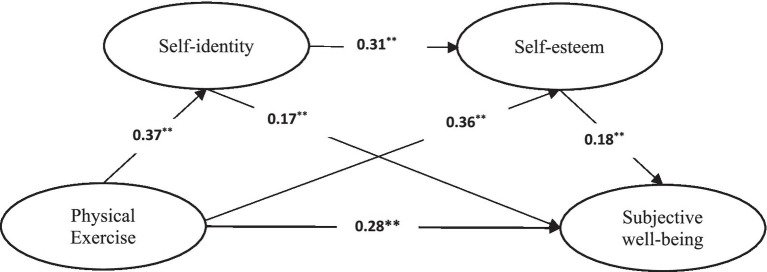
Chain mediating model of self-identity and self-esteem between physical exercise and subjective wellbeing.

The test was performed using the bootstrap method (repeated sampling 5,000 times). As depicted in Table 3, the mediating effect is as follows: The confidence interval for physical exercise → self-identity → subjective wellbeing was [0.04, 0.09], with an intermediary effect size of 0.06. The total effect accounts for 42.86%. The confidence interval for physical exercise → self-esteem → subjective wellbeing was [0.04, 0.09], with an intermediary effect size of 0.06. The total effect accounts for 42.86%. The independent contributions of self-identity and self-esteem each account for more than 40% of the total effect, indicating that physical exercise can not only directly enhance subjective wellbeing through self-identity but also have a direct impact through self-esteem. Together, self-identity and self-esteem account for 85.72% of the total effect, far exceeding the 50% threshold. This indicates that physical exercise influences subjective wellbeing through the psychological mechanism of “self-identity → self-esteem” rather than through direct action. As two independent yet complementary psychological resources, the combined effect of self-identity and self-esteem is much greater than that of a single variable. The confidence interval for physical exercise → self-identity → self-esteem → subjective wellbeing was [0.01, 0.03], with a mediating effect size of 0.02. Although the chain mediating path accounts for only 14.28% of the total effect, it reveals the progressive relationship of “self-identity → self-esteem,” providing a dynamic perspective for understanding the accumulation of psychological resources. The confidence interval does not include 0, which indicates that the mediation effect is significant. Figure 2 illustrates the mediating effects of self-identity and self-esteem on physical exercise and subjective wellbeing.

**Table 3:** Mediating effects and effect sizes.

Path	Effect size	Mediator proportion of total effect	95% Confidence interval
Physical exercise → Self-identity → Subjective well-being	0.06	0.06/0.14 = 42.86%	0.04–0.09
Physical exercise → Self-esteem → Subjective wellbeing	0.06	0.06/0.14 = 42.86%	0.04–0.09
Physical exercise → Self-identity → Self-esteem → Subjective wellbeing	0.02	0.02/0.14 = 14.28%	0.01–0.03
Total effect	0.14		0.10–0.20

## 4 Discussion

### 4.1 Physical exercise and subjective wellbeing

Extensive research has shown that physical exercise has a direct impact on college students’ subjective wellbeing and can also influence it through other mediating variables. Physical exercise plays a crucial role in promoting college students’ subjective wellbeing and enhancing their happiness index. The endorphin hypothesis theory suggests that engaging in physical exercise of a certain intensity maintains high levels of endorphins in the body, which can invigorate the exerciser, induce feelings of pleasure, and have significant value in improving negative emotions and enhancing psychological health. Based on the endorphin hypothesis theory, the release of endorphins during physical exercise by college students induces feelings of pleasure, and maintaining high levels of pleasure contributes to increasing their levels of subjective wellbeing, positively impacting the overall evaluation of individuals and their lives (Beiter et al., 2015).

The goal theory of subjective wellbeing suggests that it is closely related to human needs and intrinsic motivations. Subjective wellbeing can be enhanced when an individual’s goals align with their intrinsic needs. The self-determination theory of subjective wellbeing also emphasizes that the fulfillment of autonomy, competence, and relatedness is a key factor for subjective wellbeing. When college students engage in physical exercise based on their intrinsic needs, the sense of achievement and satisfaction they experience can enhance their subjective wellbeing. Additionally, through physical exercise, college students facilitate communication and interaction with others and society, thereby establishing good social relationships and fulfilling their relatedness needs. By striving and collaborating with others in physical exercise, college students can achieve progress and accomplishments, gaining recognition and respect from others, thereby satisfying their competence needs. Therefore, there is a positive correlation between physical exercise and subjective wellbeing among college students; the higher the level of physical exercise, the higher their subjective wellbeing.

### 4.2 The independent mediating role of self-identity

In a healthy and active lifestyle, college students need to possess excellent athletic skills and strong psychological qualities during physical exercise. When they successfully complete exercise tasks, they fully affirm their performance, have a clearer understanding of their psychological resilience and academic life status, achieve holistic development, and thus enhance their sense of self-identity (Sánchez-Miguel et al., 2017). A previous study divided identity into self-identity and social identity and showed that physical exercise supports an individual’s psychological resilience and athletic ability, thereby achieving self-identity through comprehensive personal development (Verkooijen and de Bruijn, 2013). According to Erikson’s theory of personal self-development, adolescence is characterized by a coexistence of confusion and identity, with the primary task being the development of self-identity. In this stage, college students with well-developed self-identity possess strong beliefs and correct values, can objectively recognize themselves, view things dialectically, accurately, and stably accept themselves and their surroundings, and experience a more positive sense of wellbeing. Conversely, college students with confused self-identity are easily influenced by others and the environment, exhibit more uncertainty and non-acceptance in their self-perception, are reluctant to face problems directly, and cannot accept criticism or failure, resulting in a more negative experience of wellbeing (Hofer et al., 2011). The formation of self-identity can enhance an individual’s self-efficacy and improve their adaptability, thereby influencing their psychological wellbeing and enhancing subjective wellbeing (Chen and Yao, 2010). Currently, there is extensive research on the pairwise relationships between physical exercise, self-identity, and subjective wellbeing, but limited research on the role of self-identity between physical exercise and subjective wellbeing, and almost no research on the direct relationship mechanism among the three. This study constructs a mediation model, and the results from the mediation effect testing indicate that self-identity mediates the relationship between physical exercise and subjective wellbeing, with a significant mediating effect. This evidence indicates that physical exercise can directly influence subjective wellbeing and can also influence subjective wellbeing through the mediating role of self-identity.

### 4.3 The independent mediating role of self-esteem

Firstly, physical exercise is positively correlated with self-esteem, and regression analysis further shows that physical exercise can significantly predict college students’ self-esteem (Kim and Ahn, 2021). Adolescents’ self-esteem consists of various specific components, forming a multidimensional hierarchical structure. To improve overall self-esteem levels, it is necessary first to implement interventions that enhance self-esteem in specific areas. Physical self-esteem, as a specific domain of overall self-esteem, plays a key role in influencing adolescents’ overall self-esteem levels. Physical exercise is the most effective and direct method to improve physical abilities; therefore, it can enhance physical self-esteem and subsequently improve an individual’s overall self-esteem (Auttama et al., 2021). By actively participating in physical exercise, college students can directly experience significant feelings of success and achievement. These positive experiences effectively boost their self-confidence. Participating in team sports makes it easier for college students to earn respect and recognition from others, thereby achieving notable psychological benefits and further enhancing their self-esteem.

Secondly, self-esteem is positively correlated with subjective wellbeing. As an inherent psychological resource, self-esteem has a positive impact on subjective wellbeing. College students with higher levels of self-esteem often possess a stronger ability to perceive happiness, hold more positive evaluations of themselves, recognize the positive aspects of life, and are more likely to feel satisfied and happy. They are better equipped to cope with life’s challenges and difficulties, maintain an optimistic outlook, and thereby experience more positive emotions (Garcia Meneguci et al., 2021). This positive psychological state makes it easier for them to experience happiness, resulting in higher levels of subjective wellbeing.

Furthermore, to understand the intrinsic relationship among physical exercise, self-esteem, and subjective wellbeing, we constructed a mediation model. The mediation effect testing revealed that self-esteem mediates the relationship between physical exercise and subjective wellbeing, consistent with previous research findings. This indicates that physical exercise can directly enhance college students’ subjective wellbeing and also indirectly improve it through the mediating role of self-esteem (Kim and Ahn, 2021).

### 4.4 The chain mediating role of self-identity and self-esteem

This study constructed a structural model with self-identity and self-esteem as mediating variables to examine the impact of physical exercise on subjective wellbeing. The results showed that self-identity and self-esteem served as a chain mediating mechanism between physical exercise and subjective wellbeing. This indicates that self-identity can directly impact college students’ subjective wellbeing and also influence it through the chain mediation of self-identity and self-esteem.

Firstly, physical exercise is positively correlated with self-identity; the higher the level of physical exercise, the higher the degree of self-identity among college students (Sánchez-Miguel et al., 2017). Through physical exercise, college students can not only improve and promote the development of physical and mental health but also experience personal growth and changes during the process. This experience helps individuals develop positive self-cognition and gain a clearer understanding of their abilities and values, thereby enhancing their self-identity.

Secondly, self-identity is significantly positively correlated with self-esteem. College students’ self-identity directly predicts their level of self-esteem; the higher the sense of self-identity, the higher the level of self-esteem. College students who have a clear and acknowledged self-perception often hold more positive self-perceptions. This positive self-cognition can enhance their self-esteem, making them more confident and self-respecting. Additionally, self-identity helps individuals maintain a tenacious attitude in facing challenges and difficulties, which further supports their ability to preserve and enhance their self-esteem.

Furthermore, self-esteem significantly and positively predicts subjective wellbeing; the higher the level of self-esteem, the stronger the individual’s subjective wellbeing (Du et al., 2017). College students with higher self-esteem levels are usually more confident and optimistic and can cope more actively with various life situations. They are more likely to perceive the beauty and happiness in life and maintain a more positive attitude toward life’s challenges and difficulties. Therefore, individuals with higher levels of self-esteem often possess stronger subjective wellbeing.

In summary, self-identity and self-esteem serve as a chain mediating mechanism between physical exercise and subjective wellbeing. Physical exercise influences an individual’s self-identity, which then boosts their self-esteem and ultimately has a positive impact on subjective wellbeing. This mechanism reveals the profound influence of physical exercise on psychological health and offers theoretical and practical guidance for enhancing individuals’ subjective wellbeing.

### 4.5 Research value

This study presents a new perspective on the chain-mediating role of self-identity and self-esteem between physical exercise and subjective wellbeing, unlike the typical single mediating variable observed in previous studies. Instead, it explores the impact on subjective wellbeing through the combination of multiple mediating variables, enriching the existing theoretical framework. This research not only confirmed the direct positive impact of physical exercise on subjective wellbeing but also revealed, through the chain mediating effect of self-identity and self-esteem, how physical exercise indirectly influences the subjective wellbeing of college students through these psychological mechanisms. This study provides a more comprehensive understanding of the positive effects of physical exercise on mental health. Compared with the parallel mediation model, this study confirmed the significant predictive role of self-identity on self-esteem, revealed the hierarchical transmission relationship among variables, and deepened the understanding of the core development path of “identity → self-evaluation → psychological wellbeing.” This model offers a new perspective for optimizing mental health intervention strategies for college students. Using a composite intervention model of “physical exercise + self-identity construction,” it promotes a progressive enhancement effect from identity confirmation to self-worth improvement, providing new intervention strategies for schools and educational institutions.

### 4.6 Limitations and future directions

Although this study confirmed that self-identity and self-esteem have a chain-mediating effect between physical exercise and subjective wellbeing, there are still some limitations. First, the use of self-report surveys might introduce certain social desirability biases, which could affect the rigor of the study. In the future, data collection could combine external evaluations with self-reports. Second, the study employed a cross-sectional research method to explore the relationship mechanism between physical exercise and college students’ subjective wellbeing. However, this method does not infer causal relationships between variables. Future studies could adopt longitudinal tracking or experimental intervention designs for more in-depth research. Additionally, the participants in this study were selected from a single university. The uniqueness of the sample source may limit the universality of the conclusion. The student group has a relatively high degree of homogeneity in terms of family socioeconomic status, cultural values, and educational stressors. While this homogeneity helps to control interfering variables, it may also lead to the amplification or weakening of variable relationships by specific cultures or environments, thereby limiting the external validity of the conclusion. Future research should focus on various types of universities across different regions, systematically analyze the moderating effects of cultural values and educational systems on the model path, and further verify the validity of the conclusions of this study.

## 5 Conclusion

Based on data analysis and discussion, this study elucidated the mechanism of the relationship between physical exercise and subjective wellbeing. It has significant practical implications for enhancing the subjective wellbeing of college students and reaches the following conclusions: (1) Physical exercise is significantly positively correlated with subjective wellbeing. (2) Self-identity independently mediates the relationship between physical exercise and subjective wellbeing. (3) Self-esteem independently mediates the relationship between physical exercise and subjective wellbeing. (4) Self-identity and self-esteem together act as a chain of mediators between physical exercise and subjective wellbeing.

## Data Availability

The raw data supporting the conclusions of this article will be made available by the authors, without undue reservation.
