# Supplementary material for: Relationship between physical exercise and subjective wellbeing in university students: the chain mediation role of self-identity and self-esteem
Source: Front Psychol. 2025 Sep 3;16:1637779. doi: 10.3389/fpsyg.2025.1637779 (PMC12440742; doi:10.3389/fpsyg.2025.1637779)
Supplement: Supplementary file 1 [file Supplementary_file_1.docx]

# Appendix

# Questionnaire on Physical Activity and College Students' Subjective Well-Being

Dear Student,

Hello! This questionnaire will take approximately 3 minutes of your time. The purpose of this survey is to understand the relationship between physical activity and college students' subjective well-being. The survey results will be used solely for scientific research. There are no right or wrong answers, so please feel free to select the answers that best reflect your actual situation. Thank you for your support and cooperation! Wishing you success in your studies and happiness in your life!

**I. General Information Survey**

1. Your gender:
2. ① Male ② Female
3. Your age: ( ) years
4. ① 18 ② 19 ③ 20 ④ 21 ⑤ 22
5. Your current year of study:
6. ① Freshman ② Sophomore ③ Junior ④ Senior

**II. Physical Activity Scale**

(Likert 5-point scale: Strongly Disagree, Disagree, Neutral, Agree, Strongly Agree; scored 1-5)

1. It is difficult for me to stop exercising.
2. After a few days without exercise, I have a strong desire to engage in physical activity.
3. I find it hard to accept a lifestyle without physical exercise.
4. Physical exercise is an indispensable part of my life.
5. I am able to consistently maintain my physical exercise routine.
6. I rarely interrupt my physical exercise and can sustain it over the long term.
7. I have a habit of exercising regularly.
8. I frequently participate in physical activities.

**III. Self-Identity Scale**

(Likert 6-point scale: "Not at all" 1 point, "Not very much" 2 points, "Somewhat not" 3 points, "Somewhat yes" 4 points, "Quite yes" 5 points, "Completely yes" 6 points)

1. I am striving to achieve my personal goals.
2. I have things that I am particularly passionate about.
3. I know what kind of person I am, with my own hopes and pursuits.
4. I have a clear idea of what I want to do in life.
5. I have made significant decisions regarding my own life.
6. I have seriously thought about what kind of person I am and what I should do.
7. I have questioned the lifestyle expected of me by my parents or people around me.
8. I have never lost confidence in the worldview I hold for myself.
9. I am actively seeking out things that I can dedicate myself to.
10. I take different situations seriously.
11. Regarding what kind of person I am and what I am capable of, I am comparing several possible options and seriously considering these issues.
12. I believe that I can do meaningful things in my life.

**IV. Self-Esteem Scale**

(Likert 5-point scale: "Strongly Disagree" 1 point, "Disagree" 2 points, "Neutral" 3 points, "Agree" 4 points, "Strongly Agree" 5 points)

1. I feel that I am a valuable person.
2. I feel that I possess many good qualities.
3. I tend to view myself as a successful person.
4. I am capable of doing things well, just like most people.
5. I feel that I have qualities I can be proud of.
6. I have a positive attitude toward myself.
7. Overall, I am satisfied with myself.
8. I often hold myself in high regard.
9. I do not feel that I am useless.
10. I do not think that I am good for nothing.

**V. Subjective Well-Being Scale (Likert 7-point scale)**

**(1) Which number value below best describes your feelings about life? Please mark the appropriate number that corresponds to your situation.**

| Description | **Scale** | | | | | | | Description |
| --- | --- | --- | --- | --- | --- | --- | --- | --- |
| Bored | **1** | **2** | **3** | **4** | **5** | **6** | **7** | Interesting |
| Painful | **1** | **2** | **3** | **4** | **5** | **6** | **7** | Happy |
| Useless | **1** | **2** | **3** | **4** | **5** | **6** | **7** | Valuable |
| Lonely | **1** | **2** | **3** | **4** | **5** | **6** | **7** | Have many friends |
| Empty | **1** | **2** | **3** | **4** | **5** | **6** | **7** | Fulfilled |
| Hopeless | **1** | **2** | **3** | **4** | **5** | **6** | **7** | Hopeful |

(2) How satisfied or dissatisfied are you with life overall? Which number value best describes your level of satisfaction or dissatisfaction?

| Depressed | **1** | **2** | **3** | **4** | **5** | **6** | **7** | Rewarded |
| --- | --- | --- | --- | --- | --- | --- | --- | --- |
| Life has given me no opportunities | **1** | **2** | **3** | **4** | **5** | **6** | **7** | Life has been too good to me |
